# Community pharmacists’ knowledge, perceptions, and practices about topical corticosteroid counseling: A real-world cross-sectional survey and focus group discussions in Korea

**DOI:** 10.1371/journal.pone.0236797

**Published:** 2020-07-29

**Authors:** Min Jung Kang, Ji Hyun Park, Sunny Park, Nam Gyu Kim, Eun Young Kim, Yun Mi Yu, Do Young Kim, Ju-Yeun Lee, Wan Gyoon Shin, Soo An Choi

**Affiliations:** 1 Department of Pharmacy and Biomedical Research Institute Clinical Trials Center, Seoul National University Hospital, Seoul, South Korea; 2 College of Pharmacy, Korea University, Sejong, South Korea; 3 College of Pharmacy and Research Institute of Pharmaceutical Sciences, Korea University, Sejong, South Korea; 4 Clinical Development Team, Wellmarker Bio Co, Seoul, South Korea; 5 College of Pharmacy, Chung-Ang University, Seoul, South Korea; 6 Department of Pharmacy and Yonsei Institute of Pharmaceutical Sciences, College of Pharmacy, Yonsei University, Seoul, South Korea; 7 Department of Dermatology and Cutaneous Biology Research Institute, Yonsei University College of Medicine, Seoul, South Korea; 8 College of Pharmacy and Research Institute of Pharmaceutical Sciences, Seoul National University, Seoul, South Korea; 9 Department of Internal Medicine, Korea University Guro Hospital, Korea University, Seoul, South Korea; University of South Carolina College of Pharmacy, UNITED STATES

## Abstract

Topical corticosteroids (TCs) are widely used to treat dermatological conditions such as eczema and psoriasis. It can be a safe and effective treatment when used appropriately. However, misguided information and corticosteroid phobia appear to contribute to inadequate adherence to therapy, leading to unsatisfactory treatment outcomes. Therefore, community pharmacists (CPs) are in a prime position to inform patients about the appropriate use of medicine. The aim of this study was to examine how the knowledge and perceptions of CPs, as well as other factors, associate CPs’ patient counseling practice around the use of TCs. A structured, validated questionnaire was distributed to CPs in the Republic of Korea, and additional focus group discussions were implemented to obtain a deeper understanding of the survey findings. We analyzed the survey results by applying a modified knowledge-perception-practice model. In addition, we used path analysis to validate the model and assessed how knowledge level and perceptions of barriers affect CPs’ counseling behavior. We ran a multiple regression to identify factors that associate CPs’ practice levels. A total of 1018 surveys were analyzed. In general, respondents had sufficient knowledge to provide appropriate patient counseling on TC use. An increase in knowledge level positively associated the quality of practice, and more knowledge increased the perception of barriers that negatively associated patient counseling. Location in rural areas and pharmacists’ perception of counseling barriers negatively associated the quality of practice. A higher level of knowledge, training in ADEs, higher proportion of OTC TC sales, and increased time for counseling positively associated the quality of practice. Therefore, minimizing barriers such as negative perceptions is very important in facilitating CPs’ counseling practice around TC use.

## Introduction

Topical corticosteroids (TCs) are used in a variety of dermatological diseases, ranging from mild eczema to severe psoriasis. Despite TCs having clear therapeutic value, misconceptions and concerns regarding adverse drug events (ADEs) related to TCs are widespread [1, 2]. Attitudes of patients towards TCs appear to vary. Some underuse TCs out of fear of ADEs, whereas others overuse or misuse them. Such negative perceptions appear to play a role in patients’ inadequate adherence to therapy, which leads to unsatisfactory treatment outcomes [3].

Despite TCs being topical agents, cases of ADEs related to their use have been well documented in the literature [4–11]. The risk factors for ADEs include steroid potency, delivery vehicle, application frequency, duration of treatment, and incorrect usage [12, 13]. Additionally, multiple TC classification systems are currently used, and how TCs should be categorized is ambiguous. Further, the range of TC agents available by prescription and over-the-counter (OTC) differs by country. A seven-group classification system of TCs is used in South Korea, and a variety of OTC TCs are available, including prednisolone and dexamethasone single agents, and combination agents containing betamethasone and gentamicin. In this situation, pharmacists will play an important role in the correct usage of TCs by Koreans, which will help to reduce the related ADEs.

A basic understanding of several key factors should help clinicians select appropriate preparations that maximize therapeutic efficacy and minimize the potential for adverse effects. Previous studies have investigated how doctors prescribe TCs and how nurses educate patients on using TCs for treatment [14–18]. However, research on how pharmacists counsel patients on the use of TCs is scarce. Pharmacists can be the initial source of information for patients regarding minor dermatological diseases. They are well positioned to recommend appropriate OTC treatments and can play a critical role in promoting the correct use of TCs. The existence of various perceptions about TCs and the availability of OTC drugs emphasize the importance of the role of pharmacists in the day-to-day healthcare environment.

Although pharmacists are important role in patient's safe and appropriate use of TCs, research on the pharmacist's counseling behavior for TCs is very lacking [19]. Such study can provide a foundation for improving the quality of counseling offered by pharmacists. Therefore, the aim of this study was to examine how community pharmacists’ knowledge, perception, and other factors associate their counseling around the use of TCs, and to explore how pharmacists can promote patients’ safe and effective use of TCs.

## Material and methods

### Design

This was a mixed-method design, comprised of a cross-sectional survey followed by focus group discussions (FGDs) to obtain a deeper understanding of the survey findings.

#### Cross-sectional survey

The first phase of the research was a cross-sectional survey.

*Questionnaire development*. A knowledge-attitude-practice (KAP) model is used to assess what is known, believed, and done about various subjects relating to health. The model fundamentally assumes that practices can be affected by attitudes formed by altering knowledge or awareness [20]. The knowledge component examines what people know and are aware of, the attitude component examines what they feel or perceive, and the practice component examines their behavior [21]. Perception was substituted for attitude, and the modified knowledge-perception-practice (KPP) model was applied throughout this study.

**Fig 1:**
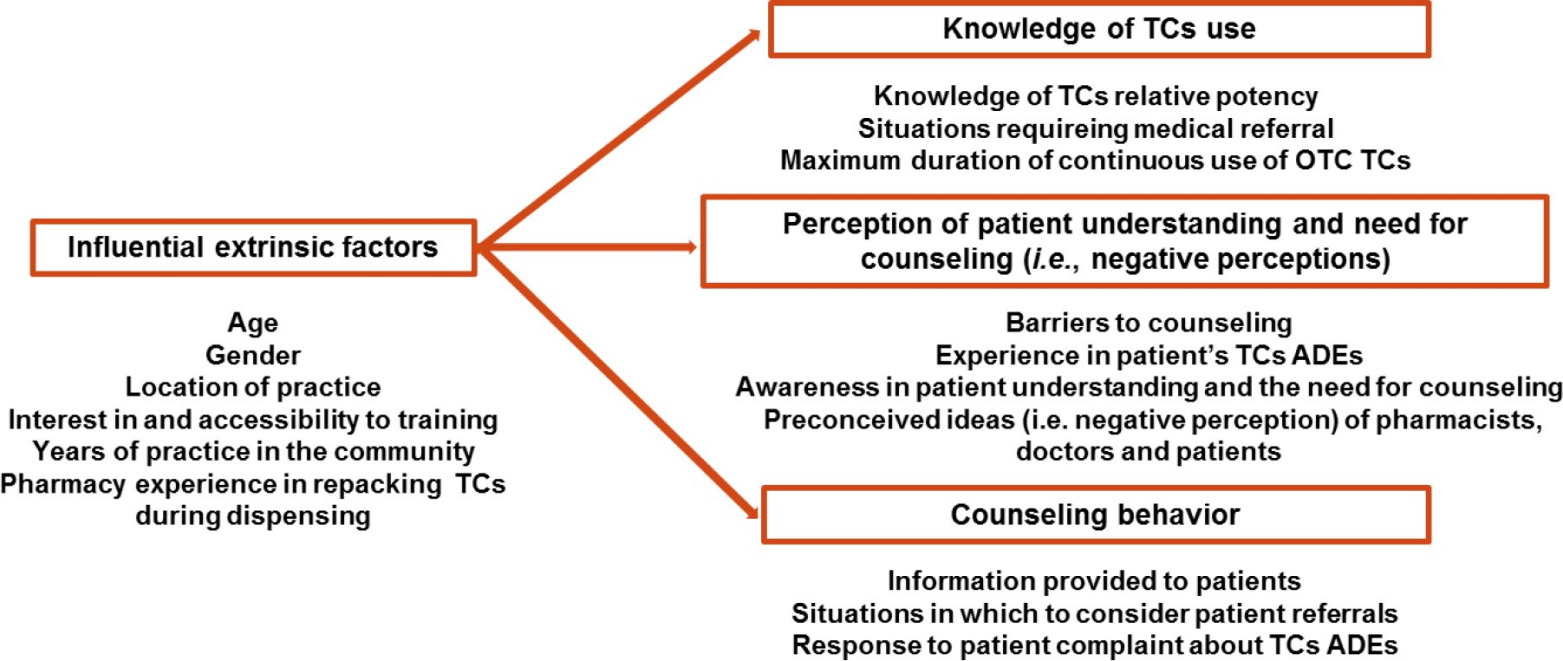
Modified knowledge-perception-practice approach to assessing TC counseling practices.

The KPP model was employed in constructing the questionnaire (Fig 1). Knowledge and awareness questions aimed to assess CPs’ level of knowledge regarding the use of TCs. Perception questions attempted to reveal CPs’ preconceived ideas regarding the use of TCs. Practice questions aimed to gather information on their counseling behavior.

A draft questionnaire was developed after an extensive review of literature on TC use and patient counseling practices [1, 16, 22]. It was reviewed for face and content validity by a panel of 11 experts, including six community pharmacists, two hospital pharmacists, and three university professors in pharmacy, and then further refined per their suggestions. The developed survey was piloted in a sub-sample of study participants. An acceptable level of reliability was reached with Cronbach’s alpha above 0.7 for all 3-point Likert scale questions measuring respondents’ counseling behavior. In the end, the distributed survey consisted of 22 questions covering the four domains listed below and took about 10 minutes to complete. A combination of numeric and text open-ended questions; single, binary, and multiple choice questions; short answer questions; ranking questions; and a set of 3-point Likert scale questions were included in the survey.

- Respondent demographics and dispensing and sales of TCs
- Patient counseling on TC use
- Perception of patient understanding of TC use
- TC-related ADEs reported to community pharmacies

Amongst the 22 questions, three questions assessed pharmacists’ level of knowledge about TC use. A multiple choice question asked CPs to make judgments about situations that require medical referrals. Results were scored between 0 to 3 points according to the number of situations correctly identified. The second short answer question asked about the maximum duration of continuous use of OTC TCs. Those who answered “14 days or fewer” scored 1 point and “more than 14 days” 0 points [23]. The last question asked respondents to rank TCs by their relative potency. Ranging from zero to five, the total knowledge score was calculated, and the respondents were divided into high (≥3 points) and low (<3 points) knowledge groups. Also, scores for CPs’ practice levels were calculated from question. Respondents rated their usual counseling practices on a Likert scale. Respondents received one point for choosing “Do not explain most of the time,” and two or three points for choosing “Explain half the time” or “Explain most of the time,” respectively. The sum was used as a variable that represents the practice level of CPs in the path analysis.

*Sample size calculation*. A multi-stage sampling method was used. First, the number of clinics that generate TC prescriptions was ascertained by reviewing data from the Health Insurance Review & Assessment Service–National Patient Samples. Dermatology, internal medicine, pediatrics, and urology were determined to be the top four specialties, generating more than 90% of the TC prescriptions in South Korea. It was assumed that 100% of the TC prescriptions come from the four specialties, and 75%, 12%, 7%, and 6% of each of these specialties, respectively, were included in the total sample extracted. The nationwide distribution of the four specialties was obtained from Statistics Korea [24]. The study sample was calculated by combining the relative weight each specialty area carries in TCs prescription generation with the nationwide distribution of each specialty across the country. The calculation yielded a total of 720 samples. Finally, the number of pharmacists to be recruited from each province was calculated with respect to the number of pharmacies in each region [25].

*Survey administration*. The finalized questionnaire was created as an online survey and uploaded to community pharmacy dispensary software that is one (about half of all registered pharmacies) of the most widely used in South Korea. In addition, researchers of this study visited a pharmacist training session to distribute and collect printed surveys. Written consent was obtained from the respondents before the initiation of the survey. Pharmacists self-administered the survey and participated on a voluntary basis through the banner when accessing the community pharmacy dispensary software. Participants were given an opportunity to enter a random drawing to win a prize upon completion of the survey. Responses were collected from early April to mid-July 2015.

*Data analysis*. Data from online surveys were exported to Microsoft Excel, and those from the paper-based questionnaire were entered manually. Descriptive analysis, one-way ANOVA and chi-squared tests were performed using Microsoft Excel 2016 and Statistical Package for SAS 9.4 (SAS Institute, Cary, NC). The path analysis method was applied to assess how CPs’ knowledge level and perception of barriers to counseling affect counseling behavior. The calculated knowledge score, perception of counseling barriers, and the calculated practice score were incorporated as variables in the model. The analysis revealed direct and indirect effects of knowledge level and perception of barriers on the level of practice. Finally, a multiple regression model was constructed to uncover factors that associate the CPs’ level of practice when covariates are controlled. These statistical analyses were performed using STATA/MP53 (version 14.0 for Microsoft Windows 64 [StataCorp LLC, College Station, Texas, USA]).

#### Focus group discussions

A qualitative approach was adopted in the second phase of this study [26]. The focus group topics were developed following an analysis of questionnaire responses, with the intention of providing further description and explanation of the survey. We have been recommended by the Korean Pharmaceutical Association for pharmacists who have been working at a community pharmacy for at least five years. Two focus groups were conducted face-to-face, semi-structured discussion from November to December 2015 with nine community pharmacists, five and four in each group. The time and place required for FGDs were decided by consensus method in consideration of the convenience of participants, and it was done after 8 p.m. in the evening after considering the working hours of local pharmacies. The FGDs lasted about two hours and were conducted by two moderators who participated in the study. All FGDs were audio recorded with participants’ consent and transcribed fully with as much detail as possible. FGDs continued until the researchers felt no new findings emerged. A five-step data analysis approach followed and analysed using thematic analysis: data reduction, coding, data display, conclusion display, and interpretation/verification. Two researchers coded each focus group independently, with consensus reached by discussion among the research team. One of whom was one of the authors and the other was a trained researcher with a doctoral degree. First, two researchers read the transcripts independently without the written notes. Then, the text was read again to classify important data and to highlight main content themes. Next, highlighted texts were reviewed as written notes to examine logical relationships and contradictions. After that, the text was reviewed to confirm or release relationships that emerged, and to identify preliminary themes. Lastly, each theme was reviewed, reconstructed, and synthesized into larger elements. The final themes and findings were then translated into English by the two researchers.

#### Ethics approval

Ethics approval was granted by the Seoul National University Institutional Bioethics Review Board for both the survey (E1502/001-004) and FGDs (1511/001-012).

## Results

### Cross-sectional survey

#### Respondent demographics and ratio of TC sales

**Fig 2:**
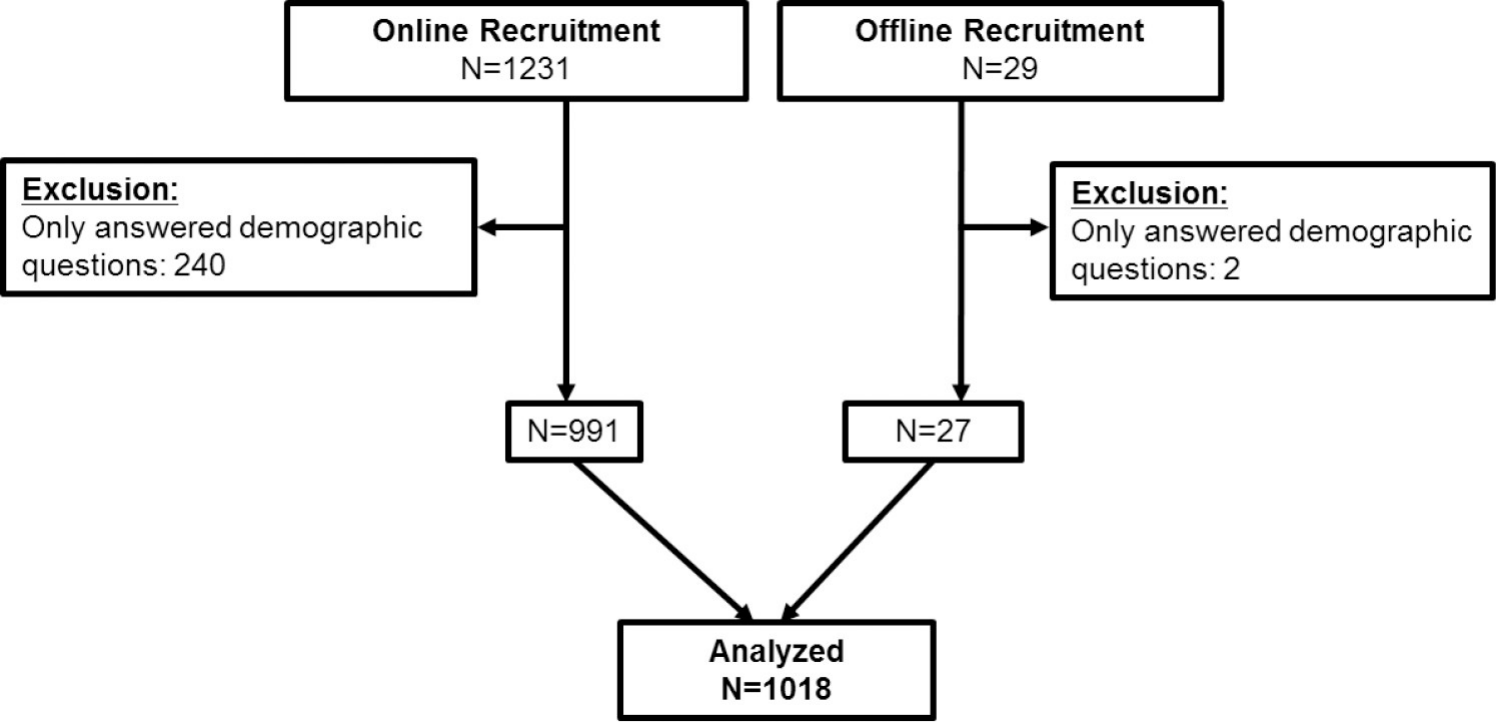
Flowchart of recruitment in the cross-sectional survey.

A total of 1260 survey responses were collected. 1231 cases of online recruitment were voluntarily submitted, accounting for approximately 12.3% of responses among 10,000 pharmacies using community pharmacy dispensary software. Of these, 1018 responses (more than 141% of the expected sample size) were analyzed (Fig 2).

More than half of the respondents (55.8%) were in their thirties and forties. Distributions between genders and amongst practice areas were relatively even. Over 62.8% of the respondents had 10 years’ experience or more in community pharmacy. More than twice as many answered that they had been trained in ADEs (68.2%) as answered that they had not been trained (31.8%). Over half of the respondents (51.2%) stated that they have repackaged TCs per prescription instructions (Table 1).

**Table 1:** Respondents’ demographic characteristics.

Characteristics	Number (%)^a^
*Age (years)*	43.69±11.63^†^
<30	130 (12.8)
30–39	258 (25.4)
40–49	309 (30.4)
50–59	228 (22.4)
≥60	91 (9.0)
*Gender*	
Male	545 (53.5)
Female	473 (46.5)
*Practice area*	
Urban	525 (51.6)
Rural	493 (48.4)
*Experience as a community pharmacist (years)*	14.66±11.00^†^
<10	379 (37.2)
10–19	299 (29.4)
20–29	216 (21.2)
≥30	124 (12.2)
*Have you been trained in ADEs*?	
Yes	694 (68.2)
No	324 (31.8)
Dispensing and sales pattern of TCs (%, monthly average)	
Prescription (part of OTC): nonprescription	58 (13.6): 42
Sales order of nonprescription TCs	
① Combination with anti-bacterials or anti-fungals	
② TC-only formulation	
③ Combination with other ingredients	
Experience repackaging* TCs during dispensing (%)	51.2

^**a**^ Total number of responses for age does not equal 1018 because two responses were missing.

^†^Mean ± standard deviation

*The dosage of prescription TCs does not correspond with that of commercial products.

#### Community pharmacists’ knowledge of TC use

Approximately one fifth of the respondents (21.9%) correctly selected five or more scenarios when asked to identify situations that require medical referrals. Most respondents (87.7%) answered “14 days or fewer” when asked about the maximum duration of continuous TC use. Over 81% of the respondents correctly ranked the three given TCs in order of decreasing potency.

There was a greater proportion of high scorers among the less experienced (< 19 years) respondents than among the more experienced. A large proportion of high scorers thought barriers to counseling exist. No statistically significant difference was seen between high and low knowledge groups with respect to training in ADEs and experience with patient complaints of ADEs after TC use (Table 2).

**Table 2:** Factors associating pharmacists’ knowledge level.

Factors		High knowledge (%)	Low knowledge (%)	*p*-value°
Experience as a community pharmacist (years)	<10	273 (84.8)	49 (15.2)	0.001
	10–19	233 (87.3)	34 (12.7)	
	20–29	148 (80.9)	35 (19.1)	
	≥30	68 (70.1)	29 (29.9)	
Training in ADEs	Trained	499 (69.1)	97 (16.3)	0.457
	Not trained	223 (30.9)	50 (18.3)	
Believe barriers to counseling exist	Yes	525 (86.2)	84 (13.8)	0.001
	No	195 (76.5)	60 (23.5)	
Have had patient complaints of ADEs after TC use	Yes	132 (84.6)	24 (15.4)	0.847
	No	577 (84.0)	110 (16.0)	

° Chi-squared tests

#### Perception of TC counseling and counseling behavior

Respondents selected “pharmacists’ verbal explanation” and “information on the Internet” as the two most important sources of information for patients. The frequency of non-prescribed OTC TC sales by pharmacist recommendation (68.5%) was reported to be more than twice that by patient self-selection (31.6%). Over 70% of the respondents stated that there are perceived barriers to counseling. Patients’ negative perception of TCs and doctors’ attitudes were selected as the two most prominent barriers (Table 3).

**Table 3:** Pharmacists’ perceived barriers to patient counseling on TC use.

Barriers to counseling	Number*
Patients’ negative feelings towards TCs	511
Doctors’ negative attitudes towards pharmacist counseling on TC use	251
Lack of counseling material/information	185
Lack of time	179
Presume patients’ already know about TCs	58
Others	39

* Multiple choice question

Almost half of the respondents (48.9%) selected verbal explanation as the most frequent form of counseling, followed by a combination of verbal explanation and written information (46.3%). On average, 2.6±2.14 minutes were spent preparing for counseling. Slightly more time was invested in counseling a patient on non-prescribed TCs (2.8±2.27 minutes) compared with prescribed TCs (2.4±1.60 minutes) (p = 0.0001).

We analyzed the relationship between pharmacists’ counseling content and their perception of patients’ understanding. Pharmacists provided the patient with an explanation of the essential items for TCs, regardless of the patient’s’ understanding. They informed patients about the pharmacological categories, the effectiveness, and the treatment duration of TCs. Most pharmacists believed patients would not be aware of the potency, ADE-related information, the correct amount to apply (*i*.*e*., fingertip units), choices of formulation for different application sites, and/or storage of remaining TCs, but they provided such information less than half the time (S1 Table).

#### TC ADEs reported to community pharmacies

Almost one-fifth of the respondents (18.5%) had encountered patient complaints of ADEs after TC use. Reported complaint frequency was 3.6%, 3.8%, and 6.3% on a monthly average for users of non-prescribed OTC TCs, prescribed OTC TCs, and prescription TCs, respectively.

Respondents ranked medication misuse and overuse as the most common causes of TC ADEs, followed by patient characteristics (*e*.*g*., age) and medication characteristics (*e*.*g*., potency). Most frequently reported TC ADEs include telangiectasia, followed by skin dryness and skin discoloration.

When patients reported TC ADEs to community pharmacies, almost all respondents directed them to stop using the TCs and seek medical attention. In addition, approximately one-third of the respondents stated they would verify that the patients used the medication as directed and recommend a re-trial of the medication after they re-educated the patients. Less than 10% of the respondents reported ADEs to regional pharmacovigilance centers. Pharmacists who had experience with TC repackaging were associated with higher frequencies of patient complaints of TC ADEs (Table 4).

**Table 4:** Frequency distribution of patient complaints of TC ADEs according to pharmacists’ repackaging experience at dispensing.

		Patient complained of TC ADEs		
Variables		Yes (%)	No (%)	*p*
repackaged TCs as per prescription during dispensing	Yes	97 (62.6)	338 (49.2)	0.003
	No	58 (37.4)	349 (50.8)	

#### Assessment of the modified KPP model

**Fig 3:**
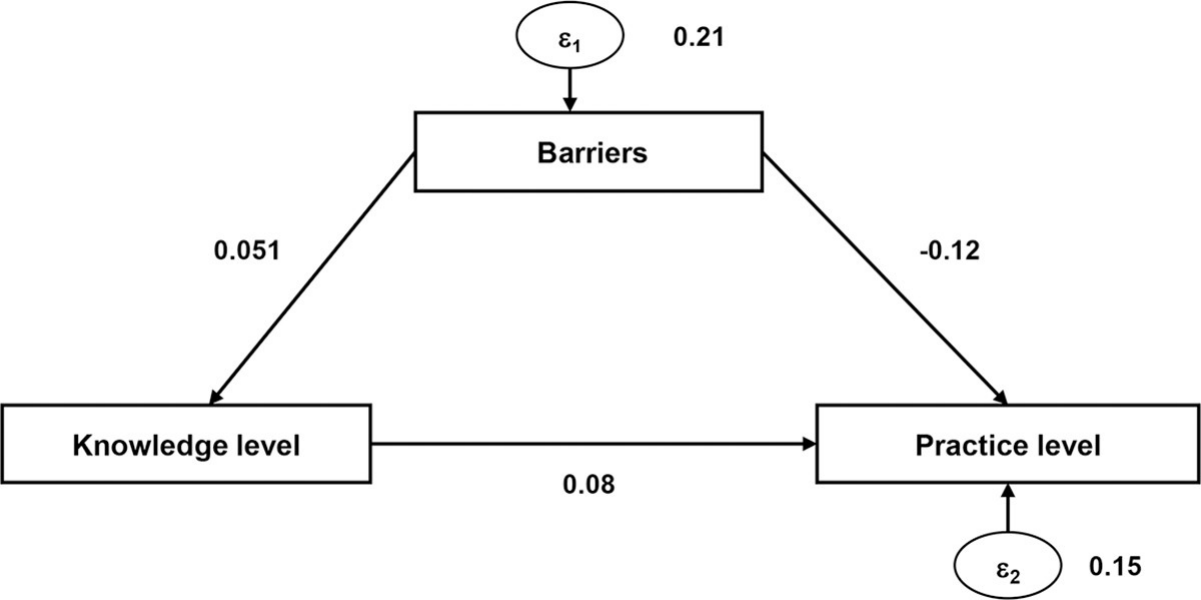
The modified KPP model used in the survey. Path analysis results showed direct and indirect effects on practice level. Knowledge level positively affected the practice level, although the increased knowledge level also increased sensitivity to perceived barriers, which negatively affected the practice level.

We conducted a path analysis on the relationship between the knowledge scores of CPs and their perception of barriers to patient counseling, which affects their counseling behavior. A higher level of knowledge positively affected the level of practice. However, the increase in knowledge level simultaneously negatively influenced the perception of counseling barriers. The total of direct and indirect effects from knowledge level and perception of barriers on the level of practice was positive (Fig 3 and S2 Table). The chi-square goodness-of-fit test value was <0.001, which suggests that the model fits the data well.

#### Factors that associate level of practice

To quantify the factors that associate the level of practice, a multiple regression was performed on the data of 843 respondents. The degree of counseling was reduced by 0.114 for pharmacists practicing in rural areas compared with those practicing in urban areas. The degree of counseling was also reduced by 0.115 for pharmacists who believe barriers exist compared with those who believe otherwise, at a statistically significant level of *p* < 0.001. Training in ADEs, increased time for patient counseling, and a higher level of knowledge of TC use were positively associated with higher degrees of counseling (Table 5).

**Table 5:** Identified factors influencing pharmacists' level of practice.

Variables	Coef.	SE	*t*-value	CI	
Age of Pharmacist	0.001	0.002	0.525	-0.003	0.005
Female Gender	0.041	0.026	1.556	-0.011	0.092
Working Area: Rural	-0.114***	0.026	-4.407	-0.164	-0.063
Experience in Community Pharmacy (Years)	-0.003	0.002	-1.401	-0.007	0.001
Training in ADEs	0.113***	0.028	4.006	0.058	0.169
A Greater Proportion of OTC TCs Selected by Patients	0.001+	0.001	1.942	0	0.003
Preparation Time for Patient Counseling (mins)	0.002	0.003	0.514	-0.005	0.008
Time Provided for Patient Counseling (mins)	0.051***	0.008	6.468	0.035	0.066
Higher Level of Pharmacists' Knowledge	0.080***	0.013	6.119	0.054	0.105
Pharmacists' Perception of Counseling Barriers	-0.115***	0.028	-4.084	-0.171	-0.06
Constant	1.846***	0.090	20.599	1.67	2.022
R-squared	0.156				

Standard errors in parentheses

*** *p*<0.001, ** *p*<0.01, * *p*<0.05, + *p*<0.10

Coef; Coefficient, SE; Standard Error, CI; Confidence Interval

### Focus group discussions

A total of three male and six female pharmacists, eight of whom had experience in community pharmacy for 10 years or more, formed two focus groups with four or five participants in each group. They included one in their 30s, six in their 40s and two in their 50s, seven of whom were working in urban areas. Among the participants, two had doctoral degrees and four had master's degrees. Discussions were focused on management approaches to promote the safe use of TCs, and realistic problems that limit the role of pharmacists. First discussed were patterns of dispensing and sales of TCs, how counseling around TC use was done, and factors that associate counseling behavior. Most comments were consistent with the survey results (S3 Table). Participants stated that the ratio of prescribed to non-prescribed TC sales is affected by local environmental factors such as the distribution of medical specialist practices. Patients rarely seek TCs themselves, and most OTC TC sales are made on pharmacists’ recommendations. CPs’ perception of patient knowledge about the use of TCs was consistent with the survey findings that CPs expect patients not to know what TCs are indicated for or where to apply them. This lack of knowledge could lead to TC misuse, which can be a major contributor to unwanted side effects. Participants pointed to several reasons that pharmacists did not actively instruct patients on TC use, including the lack of information provided in prescriptions (*e*.*g*., frequency of application, specific application site) and patients’ and medical professionals’ negative perceptions of TCs. The panelists also stressed that repackaging TCs can lead to misuse, because the labeling is lost when TCs are transferred to other containers. Secondly, a management approach was suggested for the safe use of TCs. Participants noted that improvements in education and a more collaborative relationship between pharmacists and other health care professionals are necessary to enable pharmacists to guide patients more effectively on the correct use of TCs (Table 6).

**Table 6:** Suggestions for a management plan on the safe use of TCs.

Domain	Core	Illustrative quotes
Institutional	Information on prescription and product label Improvements in packaging and specifying content	“It would be really helpful to both patients and pharmacists for the safer use of TCs if prescriptions could include key information like application area, frequency, and the suggested usage period.” (G2P3) “The expiration date should be prominently posted on the label. Also, the recommended usage period and possible deterioration after the expiration date should be included on the label.” (G1P1) “It would be great if the use of flag labels for TCs were mandatory, as in the case of eye-drops. Otherwise, pharmaceutical companies can provide those flag labels with their products.” (G2P3) “Also it would be good if the flag labels have general warnings on TC use, like ‘Do not use more than two weeks,’ ‘Apply a thin layer,’ ‘Keep this medicine away from heat and direct sunlight’.” (G1P4) “The general cautions on TC use should be listed on both the outer box and the bottle (tube) itself, so that all cautions are kept after discarding the outer packaging.” (G1P5) “Some patients want to buy a simple TC without a prescription. In this case, they don’t want to receive lengthy medication counseling. Therefore, all sufficient information should be listed or included directly on the drug tubes or bottles.” (G1P4)
	Regulations on repackaging TCs	“Pharmaceutical companies need to produce smaller package sizes of diverse TCs products.” (G2P2) “In my opinion, it is not appropriate to repackage them into a smaller size, for the sake of patient safety. (G1P4) I think the regulation should be not to break down the original package into smaller portions.” (G1P5, G2P3) “In the case of eye ointments, it is not allowed to repackage them into smaller portions. Therefore, it is okay to dispense a 20 g eye ointment tube if we don’t have the originally prescribed 15 g tubes in our formulary.” (G2P2) “I don’t know the reason for insisting on 30 g of OTC TCs. If OTC TCs are provided in smaller packages, like 5 or 10 g tubes, pharmacists might provide better counseling with sufficient information on TC use, like the expected usage periods or when to visit the doctor’s office again.” (G1P3)
Relational aspect	Consistent communication between doctor and pharmacist	“It is very important for medical professionals to provide consistent information to patients to avoid any misunderstandings. Sometimes pharmacists could unintentionally cause tension between a doctor and a patient. This is why we need to develop a protocol. Ideally, a regular meeting with doctors and pharmacists to discuss safe drug use could change the problematic condition.” (G1P5)
Educational aspect	Patient education system	“We can rarely find ointments in the pharmaceuticals disposal bins brought in from households, which is caused by a poorer understanding of the term of validity. It indicates that education is essential.” (G2P1) “In fact, after implementing an education program for the public to collect waste pharmaceuticals, we have seen a great amount of ointment products, which is good evidence of the necessity of education for the public.” (G1P4)
	Pharmacist education system	“Given the importance of consistent information, if different pharmacists provide consistent information, then the public would accept it with confidence. This speaks to the importance of developing a protocol from studies and discussion meetings.” (G1P5) “A nationwide program, such as an online training program, is needed, since the current form of offline education is clearly limited in terms of expansion.” (G1P2)

## Discussion

This study was to explore CPs’ counseling practices around TC use. In the previous study conducted by Lau WM et al, there was a significant correlation between pharmacists’ attitudes toward information provision and their self-reported counselling behavior on most topics except for those related to corticosteroid safety where less advice was given [19]. CPs in the present study also appear to have sufficient knowledge to conduct patient counseling, and they perceive themselves as important sources of information for patients. Their level of knowledge positively associates their practice, although the elevated knowledge level increases sensitivity to perceived barriers, which negatively associates the practice.

Pharmacists who participated in this study have abundant experience in local pharmacies and TCs, as demonstrated by their knowledge scores. Actual experience as community pharmacists significantly influences knowledge level. In addition, pharmacists consider themselves major sources of information about TCs, and they play a professional role in counseling patients. According to Tucker et al., even if doctors and nurses instruct patients on the correct application of topical treatments, pharmacists reinforce this message and assist patients in remembering and understanding it [27]. Pharmacists routinely encounter a minor skin conditions and believe that they can contribute to the care of patients with such skin diseases. Especially in Korea, unfavorable perceptions of TCs and the availability of OTC drugs make the pharmacist’s counseling role even more important.

Training in ADEs shows a positive effect on pharmacists’ practice level. Interestingly, being located rurally had a negative effect. Compared with those in urban areas, pharmacists in rural areas have fewer opportunities to develop their professional skills. Other influencing factors, such as increased time spent counseling patients and higher knowledge levels, demonstrated a positive effect on the practice level. It follows that pharmacists who have more knowledge and enough time for patients provide better medication counseling. Meanwhile, pharmacists’ perception of counseling barriers had a negative effect on the level of practice.

Over 70% of the study respondents stated that barriers to counseling exist. One of the barriers cited was negative patient perception of TCs. Corticosteroid phobia, a term that refers to an irrational fear of steroid use, has been well reported and described [1, 28–30]. This phenomenon is widespread amongst both patients with dermatological conditions and their caregivers [1, 31]. One study reported that up to 72.5% of parents who have children with atopic dermatitis admitted to being worried about using TCs [31]. The association between corticosteroid phobia and the reluctance to use TCs therapeutically has been well documented [32, 33]. Adding to the problem is the fact that some mistake TCs for anabolic or oral steroids [2]. Corticosteroid phobia is a multi-faceted issue that can lead to poor treatment adherence and, in turn, result in under-treatment and treatment failure.

In our study, CPs’ counseling was rather more based on stereotypes than tailored to the patient’s situation. The information most commonly offered included “the expected efficacy,” “frequency of dosing,” and “that it is a TC.” Some information about “what to do when a TC ADE occurs” and “what to do with leftover medication after treatment completion” was offered to patients. These are what the pharmacist predicts that the patient may not know well. Corticosteroid phobia may be one reason CPs provide insufficient information to patients they think lack knowledge about TCs. In addition, Raffin et al. reported that pharmacists have only moderate confidence in topical steroids [34], which may significantly impact the perpetuation of corticosteroid phobia in patients and their caregivers. Therefore, pharmacists possibly withhold explanations or information about TCs and ADEs to avoid inducing or reinforcing corticosteroid phobia.

Because TCs are applied topically, its ADEs are often limited to local reactions (*e*.*g*., telangiectasia, xerosis). A real-world safety evaluation revealed a low incidence rate of TC ADEs amongst the general public [35]. CPs are well situated to influence the outcome of pharmacotherapy as they are usually the last healthcare professional with whom patients interact before medication is administered [27]. One strategy to minimize factors that negatively influence patients’ TC use is for CPs to develop more accurate perceptions of patients before counseling them.

Another cited barrier to patient counseling was doctors’ negative perceptions. Development of tense relationships between pharmacists and physicians when pharmacists attempt to extend their role in pharmaceutical care has been documented [35]. This is supported by data showing that an increase in OTC TC sales from patient self-selection has a marginally positive effect on pharmacists’ level of practice. Since OTC TC sales do not involve doctors, pharmacists can provide better patient counseling more freely. Recently, Millard et al. reported that an inter-professional practice gap appears to exist between dermatologists and pharmacists regarding TC perceptions and counseling strategies. Collaborative education and improved communication between the two groups may be necessary to ensure that patients receive a unified, clear message about TC application and adverse effects [36]. FGDs in our study suggested that the relational aspects of regular communication between doctors and pharmacists needs to improve to promote patients’ safe use of TCs.

Pharmacists are more available for advanced pharmaceutical care when they are freed from mechanical tasks. Simplifying the dispensing process may help pharmacists find time to offer more effective face-to-face counseling to patients. Over half of the respondents in the survey stated that they have had experienced repackaging TCs per prescription instructions and that this raised concerns. Repackaging a topical preparation is not only laborious and time consuming, but interference with the original packaging gives rise to the possibility of contamination and error. When a medicine is removed from its original primary packaging, the safety, efficacy, stability, and shelf life data provided by the manufacturer are no longer retained [37]. Despite this, topical medications prescribed in quantities other than pack-size are covered by national health insurance in Korea. In addition, many pharmaceutical companies prefer to manufacture topical medications in bulk containers of 450–500 g for economic reasons. Consequently, repackaging into smaller containers occurs at pharmacies. The manufacture of TCs in quantities that are routinely dispensed would resolve this issue.

The points discussed here highlight the importance of pharmacists adequately educating patients so that patients can receive appropriate treatment. Supporting this, a study by Ahmad et al. reported that when clinical pharmacists supply patients with information about their disease condition, use of medication, and possible side effects and how to manage them, an improvement in compliance and a reduction in corticosteroid phobia results [38]. Furthermore, a study on adolescents’ perspectives on atopic dermatitis treatment reported that adolescents receive different instructions on TC use from different clinicians and seek more in-depth information about their conditions and how TCs work. Pharmacist also had an important role to inform parents of children with eczema on the appropriate use of topical corticosteroids and emollients [32]. Therefore, close collaboration between primary care providers could ensure that parents receive uniform messages.

Real-world data are defined as data relating to patient health status or to the provision of health care and are regularly collected from a variety of sources, including electronic health records, administrative and medical claims databases, patient registries, or surveys [39]. Our study sought to examine, through questionnaires and FGDs, aspects of pharmacists’ counseling practices that could affect patients’ safe use of TCs in the real world. However, it should be noted that the results of this study were obtained from a self-administered questionnaire, and, thus, self-report bias may exist. We have assessed as scored levels to minimize the measurement limits for these self-reporting practices, but this should still take into account the possibility of over-estimated quality measurements. Moreover, respondents’ claims may differ from their actual behavior. Comparing findings of this study with the results of a patient survey may complement the shortcomings of this study. While the survey in this study has the advantage of being able to collect large numbers of samples, there is concern about the possibility of selection bias because most CPs use the same pharmacy dispensary software. To minimize the concern, we have fully recruited 1.4 times more than the initial sample, and as shown in Table 1, we have conducted the analysis after confirming that the age, gender, region, and experience of the respondents are evenly distributed. For all these efforts, this study does not directly measure the outcome for the safe use of TCs, so it should not make the error of over-interpreting the results. Finally, the interpretation and application of our findings should be limited to identifying influencing factors and exploring ways to improve the counseling practices of CPs on TCs.

## Conclusions

Findings of this study indicate that, despite community pharmacists having sufficient knowledge and awareness to guide patients on the safe and appropriate use of TCs, their counseling practices are sub-optimal. Such dissonance may be due largely to barriers such as negative perceptions associated with TCs. The nexus of pharmacists, doctors, pharmaceutical companies, and through insights on pharmacist-patient dynamic presents promising avenues for future research and intervention.

## Supporting information

S1 Table Community pharmacists’ presumption on patients’ understanding and level of counseling offered to patients. (DOCX)

S2 Table Results of the path analysis in modified KPP survey. (DOCX)

S3 Table Core contents of the focus group discussions in comparison to survey findings. (DOCX)

S1 File Questionnarie English version. (PDF)

S2 File Questionnarie Korean version. (PDF)

S3 File Questionnarie analysis back dataset. (XLSX)
