# Supplementary material for: Community pharmacists’ knowledge, perceptions, and practices about topical corticosteroid counseling: A real-world cross-sectional survey and focus group discussions in Korea
Source: PLoS One. 2020 Jul 29;15(7):e0236797. doi: 10.1371/journal.pone.0236797 (PMC7390350; doi:10.1371/journal.pone.0236797)
Supplement: S1 Table — (DOCX) [file pone.0236797.s001.docx]

|  | | Do you believe that patients’ have some level of understanding? * | |  |  |
| --- | --- | --- | --- | --- | --- |
| Question variables | Degree of counseling offered | Yes (%) | No (%) | Sum | *p* |
| That it is TCs  (pharmacologic category) | Explain most of the time | 322 (69.0) | 207 (53.4) | 529 | 0.000 |
|  | Explain half the time | 112 (24.0) | 133 (34.3) | 245 |  |
|  | Do not explain most of the time | 33 (7.1) | 48 (12.4) | 81 |  |
|  |  | 467 | 388 | 855 |  |
| Expected efficacy and effectiveness | Explain most of the time | 415 (75.2) | 223 (73.6) | 638 | 0.639 |
|  | Explain half the time | 126 (22.8) | 71 (23.4) | 197 |  |
|  | Do not explain most of the time | 11 (2.0) | 9 (3.0) | 20 |  |
|  |  | 552 | 303 | 855 |  |
| Potency (Strength) | Explain most of the time | 26 (43.3) | 238 (30.0) | 264 | 0.024 |
|  | Explain half the time | 26 (43.3) | 338 (42.6) | 364 |  |
|  | Do not explain most of the time | 8 (13.3) | 218 (27.5) | 226 |  |
|  |  | 60 | 794 | 854 |  |
| Adverse drug events  [non-prescribed TCs users] | Explain most of the time | 37 (36.6) | 252 (33.5) | 289 | 0.367 |
|  | Explain half the time | 46 (45.5) | 319 (42.4) | 365 |  |
|  | Do not explain most of the time | 18 (17.8) | 182 (24.2) | 200 |  |
|  |  | 101 | 753 | 854 |  |
| Adverse drug events  [prescribed TCs users] | Explain most of the time | 61 (43.0) | 228 (32.0) | 289 | 0.035 |
|  | Explain half the time | 55 (38.7) | 310 (43.5) | 365 |  |
|  | Do not explain most of the time | 26 (18.3) | 174 (24.4) | 200 |  |
|  |  | 142 | 712 | 854 |  |
| What to do when adverse drug event occurs | Explain most of the time | 22 (41.5) | 187 (23.3) | 209 | 0.009 |
|  | Explain half the time | 18 (34.0) | 306 (38.2) | 324 |  |
|  | Do not explain most of the time | 13 (24.5) | 308 (38.5) | 321 |  |
|  |  | 53 | 801 | 854 |  |
| How much to apply  (e.g. fingertip units) | Explain most of the time | 117 (41.9) | 234 (40.6) | 351 | 0.461 |
|  | Explain half the time | 107 (38.4) | 207 (35.9) | 314 |  |
|  | Do not explain most of the time | 55 (19.7) | 135 (23.4) | 190 |  |
|  |  | 279 | 576 | 855 |  |
| Frequency of application | Explain most of the time | 245 (87.8) | 471 (81.8) | 716 | 0.054 |
|  | Explain half the time | 33 (11.8) | 97 (16.8) | 130 |  |
|  | Do not explain most of the time | 1 (0.4) | 8 (1.4) | 9 |  |
|  |  | 279 | 576 | 855 |  |
| Duration of treatment | Explain most of the time | 150 (53.8) | 316 (54.9) | 466 | 0.139 |
|  | Explain half the time | 111 (39.8) | 202 (35.1) | 313 |  |
|  | Do not explain most of the time | 18 (6.5) | 58 (10.1) | 76 |  |
|  |  | 279 | 576 | 855 |  |
| Choice of formulation for specific application site | Explain most of the time | 98 (35.1) | 189 (32.8) | 287 | 0.122 |
|  | Explain half the time | 127 (45.5) | 239 (41.5) | 366 |  |
|  | Do not explain most of the time | 54 (19.4) | 148 (25.7) | 202 |  |
|  |  | 279 | 576 | 855 |  |
| What to do with remaining TCs after the completion of treatment | Explain most of the time | 55 (34.2) | 109 (15.7) | 164 | 0.000 |
|  | Explain half the time | 53 (32.9) | 250 (36.1) | 303 |  |
|  | Do not explain most of the time | 53 (32.9) | 334 (48.2) | 387 |  |
|  |  | 161 | 693 | 854 |  |

**^a^** Total number of responses for this question add up to 854-855.
