# Supplementary material for: Community pharmacists’ knowledge, perceptions, and practices about topical corticosteroid counseling: A real-world cross-sectional survey and focus group discussions in Korea
Source: PLoS One. 2020 Jul 29;15(7):e0236797. doi: 10.1371/journal.pone.0236797 (PMC7390350; doi:10.1371/journal.pone.0236797)
Supplement: S2 Table — (DOCX) [file pone.0236797.s002.docx]

| Hypothesized path relationships | | Path (beta) | t | p |
| --- | --- | --- | --- | --- |
| Direct effects | Knowledge level → Barriers | 0.051 | 3.23 | 0.001* |
|  | Barriers → Practice level | -0.123 | -4.21 | <0.001* |
| Indirect effects | Knowledge level → Practice level | 0.080 | 5.90 | <0.001* |
| Total effects | Barriers ↓  Knowledge level → Practice level | 0.074 | 5.41 | <0.001* |

* p≤0.001
