# Supplementary material for: Community pharmacists’ knowledge, perceptions, and practices about topical corticosteroid counseling: A real-world cross-sectional survey and focus group discussions in Korea
Source: PLoS One. 2020 Jul 29;15(7):e0236797. doi: 10.1371/journal.pone.0236797 (PMC7390350; doi:10.1371/journal.pone.0236797)
Supplement: S3 Table — (DOCX) [file pone.0236797.s003.docx]

| Domain | Core | Illustrative quotes |
| --- | --- | --- |
| Dispensing & sales | Differences in  sales proportion of OTC and  prescription TCs | “It varies by the type of pharmacy stores. For example, if a pharmacy store …located nearby dermatology clinic, then the frequency of steroid ointment sales would be higher. If a pharmacy is located in residential area or mainly deals with general OTC drugs… a large proportion of sales are by recommendation of pharmacist.” (G1P2). |
| Selection of  products | Self-selection vs.  pharmacist recommendation | “Amongst all …about 80% are recommended by pharmacist based on information gathered from patient counseling, …. However, majority of the 20% are the cases where patients are previously recommended the drug by a pharmacist, and they are repurchasing it since they got better after using the drug. They do not purchase the drug based on the information on ads.”(G1P1) |
|  | Advertising’s impact on  Selection | “There are not many steroid products being advertised, and this would have led to the lower purchase frequency by patients’ own choice.” (G1P3) |
| ADE experience & patient use behavior | Knowledge on how to use TCs  Misuse and  overuse | “Speaking of misuse, generally, patients do not have an appropriate understanding about the application site. Therefore, they apply it on the face and later use it on foot and other parts of their body. Moreover, side effects are expected when all the family members share the same steroids, and also when they apply it on itchy skin and pimples.” (G1P4)  “Patients tend to think that topical medicine including ointment is less likely to be absorbed compared to orally taken drugs and thus take less care.” (G2P1)  “In reality, there are patients who apply steroid ointments on wounds.” (G2P1)  “Once a patient asked me if using occlusive dressing would improve the effect of the steroid ointment. This means that he has some misunderstanding.” (G2P1)  “Most of the abuse cases are in elderly people who have used it for a long time. Thinking that all ointments are similar, family members share prescribed ointment including antifungal drugs.” (G2P1)  “People think that the effect of an ointment would last regardless of the expiry date if they keep it in a refrigerator. I think there could be far more reasons that could cause misuse and abuse of TCs beyond the cases that we have reviewed.” (G1P4) |
| Counseling status | Counseling material to  maximize the  efficiency | “Development of counseling protocol and making use of it can make medication counseling very efficient in terms of time and usefulness. For example, any product with steroids as ingredient, advisory label is attached.” (G2P1, G1P3)  “Normally, when we evaluate the quality of patient counseling on medication, we focus  on the amount of information provided rather than the quality of it. If we use well  developed counseling protocol the amount of time used for medication counseling is  less critical in judging the quality of counseling. Additionally, the characteristics of a  patient, whether it is first visit or revisit, the required amount of time for counseling would  be different.” (G2P3) |
|  | Patient counseling contents (pharmacologic category, potency,  application site,.etc.) | “In fact, steroids can be very useful and beneficial when misuse and abuse are strictly controlled by providing adequate counseling. Some pharmacists might have a negative perception towards steroids. However, I think pharmacists need to be different to non-health care professionals.” (G1P2)  “It would be better to let patients know that it is steroids so that they recognize it is different to other anti-inflammatory agents. This could result in reducing drug misuse and abuse.” (G1P3)  “It seems clear that long term use of steroids is likely to cause side effect, therefore pharmacists should provide counseling about possible effects and side effects associated with long term use to help patients’ decision on treatment.” (G1P1)  “I provide patients with information on strength of medication - low, middle, high - regardless of whether it is steroids or not. I think patients should know whether it is steroids or not, so I print it on a label and attach it on the medication. This could trigger further conversation. If patients ask if steroids are not good then I explain pros and cons with an emphasis that an appropriate use could be very beneficial.” (G1P3)  “There could be negative impression, however, a thorough explanation in the medical counseling process would eventually contribute to the safe use of medicine.” (G1P5) |
|  | Emphasis when  counseling OTC  TCs | “Knowing that long-term use of steroids is not ideal for patients, pharmacists hesitate to repeatedly give OTC TCs to patients. We recommend not to use longer than a week since a long-term use of a steroid product is likely to cause side effects.” (G1P2) |
| Barriers to  counseling | Patients’ negative  perception towards TCs use  Repackaging TCs  as per  prescription | “At clinics, doctors seldom explain to patients that their prescription calls for steroids.” (G1P1, G1P2) “From a pharmacist’s point of view, the relationship with doctors also need to be considered when offering counseling on TCs” (G1P3) “It is clear that letting patients know it contains steroids would result in safe use of drugs. However, there are other relevant factors influencing it such as external environment, prescribing pattern, and the political relationship between doctors and pharmacists.” (G2P1)  “At clinics, it is known as anti-inflammatory, skin treating medicine. Within this context, it is hard for pharmacists to be straightforward and explain that it is steroids.” (G1P3, G2P3)  “In case a prescription is written by a doctor whose specialty is not dermatology, pharmacists are reluctant to make clear that it is steroids. In that situation, they recommend not to use it for a long time and use small amounts at a time.” (G1P2)  “When the label of a TCs are removed, it is hard to figure out the expiry date. In addition, many products are commercially available in bulk. As a consequence there are occasions where pharmacists have to transfer a portion of it to a smaller container and repackage the medication. In such situation, patients will lose the original label and pharmacists are concerned about the sanitation and safety. This is a representative example of current practice that could cause mediation misuse and abuse.” (G2P3) |
|  | Lack of information relating to patients | “Limited information is available on prescription - only the name of the drug and the quantity. It is hard for pharmacists to find out for what purpose it is needed. If a pharmacist asks in detail during counseling, patients may feel anxious, which may be a problematic situation for pharmacists.” (G1P4)  “Without knowing which part of the body TCs is to be applied, medication counseling becomes a difficult job.” (G2P4) |
