# Supplementary material for: Community pharmacists’ knowledge, perceptions, and practices about topical corticosteroid counseling: A real-world cross-sectional survey and focus group discussions in Korea
Source: PLoS One. 2020 Jul 29;15(7):e0236797. doi: 10.1371/journal.pone.0236797 (PMC7390350; doi:10.1371/journal.pone.0236797)
Supplement: S1 File — (PDF) [file pone.0236797.s004.pdf]

Documents to offer to research participants (offline survey)

A STUDY TO INVESTIGATE ADVERSE DRUG EVENTS AND SAFETY ISSUES OF TOPICAL CORTICOSTEROIDS

- Survey for community pharmacists -

Greetings.

We are conducting research on behalf of the Ministry of Food and Drug Safety to establish grounds to promote safe use of topical corticosteroids.

This survey is designed to investigate **pharmacists' counseling practices with a focus on the safe use of medicine** and also to examine **adverse drug events associated with the use of topical corticosteroids** from **pharmacists' viewpoint**. Your response will remain anonymous and will only be used for research purposes.

In this survey, topical corticosteroids refer to skin preparations, excluding oral and ophthalmic agents.

This survey will take approximately **10 minutes** to complete.

We would appreciate if you could take a few moments to answer the questionnaire.

February 2015

Clinical Pharmacy Laboratory, College of Pharmacy, Seoul National University

I. Demographic characteristics and dispensing and sales of topical corticosteroids

1. **Age** ______years

2. **Gender** ☐ male ☐ female

3. **Current workplace location**

☐Seoul	☐Busan	☐Daegu	☐Incheon	☐Gwangju
☐Daejeon	☐Ulsan	☐Gyeonggi-do	☐Gangwon-do	☐Chungcheongbuk-do
☐Chungcheongnam-do	☐Jeollabuk-do	☐Jeollanam-do	☐Gyeongsangbuk-do	☐Gyeongsangnam-do
☐Jeju-do	☐Sejong			

4. **Experience as a community pharmacist (excluding experience at hospitals and pharmaceutical companies)** Total _____years

5. **Have you ever been trained in adverse drug events?**

   ☐ Yes ☐ No

Dispensing and sales of topical corticosteroids

6. **What is the sales proportion of topical corticosteroids at your pharmacy on average per month?**

   6-a. Prescription _____% **+ Non-prescription** ____% = Total 100%

   6-b. Amongst the prescribed **(Prescription** _% **+ Over-the-counter**_%**)** = Total 100%

7. **What is the proportion of sales by recommendation of non-prescription over-the-counter topical corticosteroids at your pharmacy on average per month?**

   Patient self-selection _____% **+** Pharmacist recommendation _____% = Total 100%

8. **Please rate the following non-prescription over-the-counter topical corticosteroids in order of sales frequency.**

   (Note: Give '1' to the most frequently sold agents and '3' to the least frequently sold.)

   [ ] Steroids only agents (e.g.: Lacticort Cream 1 %®)
   [ ] Steroids-antibiotics or steroids-antifungals combination agents
   (e.g.: Madecassol comp. Ointment®, Celestone-G Cream®)
   [ ] Steroids-other ingredients combination agents (e.g.: Dema-Matha Cream®)

9. **Please state three or more brand names of most frequently sold non-prescription over-the-counter corticosteroids.**

10. **Do you have the experience of <u>break open original packaging</u> and <u>dispense a portion of topical corticosteroids</u> in a separate container as given in examples below?**

Example 1: Dispense 15 g + 5 g to give prescribed 20 g when package size is 15 g.

Example 2: Dispense 10 g in a separate container as per prescription instructions when package size is 30 g.

☐ Yes (________% of all topical corticosteroids prescriptions received)

☐ No

II. Patient counseling on the use of topical corticosteroids

11. **Please answer the following questions about your patient counseling practices on the use of topical corticosteroids.**

11-a. Most frequently used counseling method

☐ Printed information ☐ Face-to-face(verbal) and printed information ☐ E-mails
☐ Face-to-face(verbal) ☐ Applications ☐ Others _______

11-b. Counseling preparation time

'Counseling preparation time' refers to the time invested in searching, reviewing, evaluating and organizing information, including verifying prescription and checking patients' past medical history before counseling a patient.

On average__________minutes per patient.

11-c. Time spent in face-to-face(verbal) counseling

On average______minutes per patient on prescription topical corticosteroids.

On average______minutes per patient on non-prescription topical corticosteroids.

12. **Please mark on items below to indicate to what extent you counsel patients using topical corticosteroids.**

		Explain most of the time	Explain half the time	Do not explain most of the time
**12-a.**	That it is **topical corticosteroids**			
**12-b.**	Expected **efficacy** and **effectiveness**			
**12-c.**	**Skin conditions** and **diseases** where topical corticosteroids **should not be used**<br>(e.g.: Do not use in the eye.<br>Do not use for non-treatment purposes such as after applying make-up or shaving.<br>Do not use in burns or frostbite of beyond second degree.)			
**12-d.**	**Strength (Potency)**			
**12-e.**	**Adverse drug events**			
**12-f.**	**What to do when adverse drug event occurs**			
**12-g.**	How to use – **dosage**<br>(e.g.: **Fingertip unit:** Amount of ointment or cream squeezed out of a tube, from the tip of an adult index finger to the first crease in the finger, which may be sufficient for a diseased site of two adult-hand size.)			
**12-h.**	How to use – **frequency of application in a day**			
**12-i.**	How to use – **duration of treatment**			
**12-j.**	How to use – **choice of formulation (ointment, cream, lotion etc.) for specific application site)**			
**12-k.**	Precautions for **storage** and **application of leftover topical corticosteroids** after treatment completion.<br>(e.g.: Do not remove medication from its original packaging and transfer to a separate container.<br>Do not use remaining medication on other conditions at patients' own discretion.)			

[Questions 13-14 are in regards to your counseling practices to assist patient self-treatment with non-prescription over-the-counter topical corticosteroids.]

13. In which of the following cases would you recommend a patient seeking non-prescription over-the-counter topical corticosteroids seeing a doctor? (multiple selection allowed)

☐ Skin lesions with effusion
☐ Skin condition of moderate or greater severity
☐ Signs of skin infection
☐ Neonates and infants (less than 24 months old) (excluding nappy rash)
☐ Applying topical corticosteroids on considerable area of body surface
☐ Others_______________________________________

14. How many days do you tell patients on non-prescription over-the-counter topical corticosteroids that it is okay to use continuously?

Maximum __________Days

15. Do you believe that there are barriers to counseling patients on topical corticosteroids?

☐ Yes (Go to Question 15-a) ☐ No (Go to Question 16)

15-a. Please select two barriers to counseling.

☐ Lack of counseling material
☐ Lack of time for counseling
☐ Doctors' negative perception towards pharmacist counseling
☐ Patients' negative perception towards topical corticosteroids
☐ Presume patients already know well about topical corticosteroids
☐ Others____

III. Pharmacists' understanding on patients' knowledge in topical corticosteroids

16. Do you think patients know to some extent on the following aspects of topical corticosteroids?

		Yes	No
**16-a.**	That it is **topical corticosteroids**		
**16-b.**	**Efficacy** and **effectiveness**		
**16-c.**	**Strength (potency)**		
**16-d.**	[Patients on **non-prescription topical corticosteroids**] **adverse drug events**		
**16-e.**	[Patients on **prescription topical corticosteroids**] **adverse drug events**		
**16-f.**	**What to do** when **adverse drug event occurs**		
**16-g.**	**Know how to use** (dosage, treatment duration, frequency of application, choice of formulation for specific application site) and comply with it.		
**16-h.**	Know how to **store** and **apply** **leftover topical corticosteroids** after treatment completion and comply with it.<br>(e.g.: Do not remove medication from its original packaging and transfer to a separate container.<br>Do not use remaining medication on other conditions at patients' own discretion.)		

17. Please select three from below that you believe are patients' main source of information about topical corticosteroids.

☐ Pharmacist's explanation ☐Product information ☐Internet ☐Friends(Acquaintances)
☐ Doctor's explanation ☐Advertisements, newspaper or expert journal ☐Information leaflet from hospitals or community pharmacies
☐ Others___________

IV. Adverse drug events of topical corticosteroids

18. Have you had patients complaining (including visits and phone calls) of adverse drug events after using topical corticosteroids at your pharmacy?

☐ Yes (Go to Question 18-a)

☐ No (Go to Question 19)

18-a. On average per month ____% of patients using **non-prescription over-the-counter** topical corticosteroids complained of adverse drug events.

18-b. **i.** On average per month ___% of patients using **prescribed over-the-counter** topical corticosteroids complained of adverse drug events.

ii. On average per month ___% of patients using **prescribed prescription** topical corticosteroids complained of adverse drug events.

19. Please rank the factors that you think are the causes of adverse drug events of topical corticosteroids. (Note: Give '1' to the most likely factor.)

[ ] Medication misuse (e.g. Use on not applicable conditions)
[ ] Medication characteristics (e.g. Strength/potency)
[ ] Patient characteristics (e.g. Elderly, with chronic diseases, neonates and infants)
[ ] Medication overuse (e.g. Patient self-treatment, not complying with treatment instructions)
[ ] Others ________________________

20. Please select three adverse drug events of topical corticosteroids that patients frequently complain.

☐ Dry skin, itchiness, irritation
☐ Bruises
☐ Acne, folliculitis
☐ Hirsutism (excessive hair growth)
☐ Systemic adverse events (including ocular symptoms)
☐ Other skin adverse events
☐ Capillary dilatation
☐ Skin atrophy, stretch marks
☐ Change in skin color (decolorization, pigmentation)
☐ Hot flashes, rosacea, perioral dermatitis
☐ Skin infection (bacterial, fungal, viral)

21. What do you do when patients on topical corticosteroids complain of adverse drug events? (multiple selection allowed)

☐ Discontinue and recommend seeing a doctor
☐ Report to regional drug safety center
☐ Check if the patient has been using topical corticosteroids as directed. Re-educate patient and recommend re-trial of the treatment.
☐ Others__________________________

22. Please rank the below three topical corticosteroids according to their strength(potency).

(Note: Give '1' to the <u>highest</u> strength(potency) agent, and give '3' to <u>lowest strength(potency)</u>.)

**Topical corticosteroids**	**Strength(potency) ranking**
**Lacticare HC lotion 1%®** (hydrocortisone 10 mg/ml, lotion)	[ ]
**Dermatop cream 0.25%®** (prednicarbate 2.5 ㎎/g, cream)	[ ]
**Dermovate ointment®** (clobetasol-17-propionate 0.5 ㎎/g, ointment)	[ ]

Thank you for completing the survey.
Your opinion will be used as valuable information contributing to improvement in public health.
