# Supplementary material for: Community pharmacists’ knowledge, perceptions, and practices about topical corticosteroid counseling: A real-world cross-sectional survey and focus group discussions in Korea
Source: PLoS One. 2020 Jul 29;15(7):e0236797. doi: 10.1371/journal.pone.0236797 (PMC7390350; doi:10.1371/journal.pone.0236797)
Supplement: S2 File — (PDF) [file pone.0236797.s005.pdf]

연구참여자에게 제공되는 서류 (오프라인상에서의 설문내용)

스테로이드외용제의 부작용 실태와 안전성 복약지도 현황 연구

- 지역약국 약사대상 설문조사 -

안녕하십니까

저희 연구진은 식품의약품안전처의 용역을 받아 스테로이드외용제의 안전사용방안 수립 근거를 마련하기 위하여 연구를 진행하고 있습니다.

본 설문지는 **스테로이드외용제를 조제·판매하는 약사**에서 **안전성 측면에서의 복약지도 현황** 및 **스테로이드외용제 부작용 실태**를 파악하기 위해 만들어졌습니다. 설문에 답변하신 내용은 무기명으로 처리되며, 연구목적 이외의 다른 용도로는 사용되지 않습니다.

본 설문에서 **스테로이드외용제**란 구강용제 및 안과용제 등을 제외한 **피부용제**만을 의미합니다.

예상 설문 소요시간은 **약 10분**입니다.

잠시 시간을 내서 설문 문항에 답해 주시면 감사하겠습니다.

2015년 2월

서울대학교 약학대학 임상약학실

I. 인구학적 특성 및 조제·판매현황

1. **연령** 만______세

2. **성별** ☐ 남 ☐ 여

3. **현재 근무지역**

☐서울특별시	☐부산광역시	☐대구광역시	☐인천광역시	☐광주광역시
☐대전광역시	☐울산광역시	☐경기도	☐강원도	☐충청북도
☐충청남도	☐전라북도	☐전라남도	☐경상북도	☐경상남도
☐제주도	☐세종시			

4. **지역약국 약사로서 근무(실무) 경력** (병원, 제약회사 등 기타경력 제외) **총 _____년**

5. **약물 부작용에 관한 연수교육을 받은 적이 있습니까?**
☐ 예 ☐ 아니오

스테로이드외용제 조제 및 판매현황

6. **귀하의 약국에서 1개월간 평균적으로 스테로이드외용제의 판매 건수의 비율은 어떻게 됩니까?**

6-a. 처방의약품 _____% **+ 비처방**의약품 ____% = 총 100%

6-b. 처방의약품 중 **(전문**의약품 _____% **+ 일반**의약품_____% **)** = 총 100%

7. **귀하의 약국에서 1개월간 평균적으로 비처방 일반의약품 스테로이드외용제 판매 시 추천비율은 어떻게 됩니까?**

환자 자가선택 _____% **+** 약사 추천 _____% = 총 100%

8. **다음 중 비처방 일반의약품 스테로이드외용제를 판매빈도순으로 번호를 부여하여 주십시오.**

(참고: 가장 많이 판매하는 제제에 '1' 부여, 가장 적게 판매하는 제제에 '3' 부여)

[ ] 스테로이드 단일제제 (예: 락티코트크림1%®)
[ ] 스테로이드-항생제 또는 스테로이드-항진균제 복합제제
(예: 복합마데카솔연고®, 쎄레스톤지크림®)
[ ] 스테로이드-기타성분 복합제제 (예: 데마메타크림®)

9. 가장 많이 판매하는 비처방 일반의약품 스테로이드외용제의 상품명을 3가지 이상 기재하여 주십시오.

____

10. 아래 예시와 같이 처방된 스테로이드외용제를 포장단위를 파괴하여 별도의 용기에 분할조제한 경험이 있습니까?

예시 1: 15g 포장단위의 스테로이드외용제가 20g 처방되어 15g+5g으로 분할조제함

예시 2: 30g 포장단위의 스테로이드외용제가 10g 처방되어 소용량 포장함

☐ 예 (스테로이드외용제 처방전 총 접수 건수의 ________%)

☐ 아니오

II. 스테로이드외용제 복약지도 현황

11. 스테로이드외용제 복약지도에 관하여 다음 물음에 답하여 주십시오.

11-a. 주된 복약지도 방법

☐ 서면 ☐ 대면(구두) 및 서면 ☐ e-mail

☐ 대면(구두) ☐ 어플 ☐ 기타 __________

11-b. 복약지도 준비시간

'복약지도 준비시간'이란 복약지도를 수행하기 전 처방검토, 환자의 약력확인 등 복약지도에 필요한 정보를 검색, 검토, 평가, 정리하는 시간을 의미합니다.

환자 1인에 대하여 **평균________분**

11-c. 대면(구두) 복약지도시 수행시간

처방의약품의 경우 환자 1인에 대하여 **평균________분**

비처방의약품의 경우 환자 1인에 대하여 **평균________분**

12. **스테로이드외용제 투약 시 복약지도하는 항목에 대하여 해당란에 표기하여 주십시오.**

		대체로 설명한다	절반의 경우 설명한다	대체로 설명하지 않는다
**12-a.**	**스테로이드외용제임**을 알림			
**12-b.**	기대되는 **효능** 및 **효과**			
**12-c.**	**사용하면 안되는** 피부 **상태** 및 **질환**<br>(예: 안과용으로 사용하지 않는다.<br>화장이나 면도 후 등 치료 이외의 목적으로 사용하지 않는다.<br>제2도 심재성 이상의 화상・동상에 사용하지 않는다)			
**12-d.**	**세기 (역가)**			
**12-e.**	**부작용**			
**12-f.**	**부작용** 발생시 **대처법**			
**12-g.**	적절한 사용 방법 – **투여량**<br>(예: **Fingertip unit**, 어른 손바닥 2개 크기의 병변에 손가락 1 마디에 해당하는 양)			
**12-h.**	적절한 사용 방법 – **1일 적정 도포 횟수**			
**12-i.**	적절한 사용 방법 – **투여 기간**			
**12-j.**	적절한 사용 방법 – **제형(연고, 크림, 로션 등)에 따른 적용부위 차이**			
**12-k.**	치료 이후 **남은 스테로이드외용제**의 **저장** 및 **적용 시** 주의사항<br>(예: 의약품을 원래 용기에서 꺼내어 다른 용기에 보관하지 않는다<br>남은 외용제를 임으로 다른 질환에 사용하지 않는다)			

[13-14번 문항은 환자의 자가치료를 돕기위해 비처방 일반의약품 스테로이드외용제 투약 시 복약지도에 관한 물음입니다]

13. 비처방 일반의약품 스테로이드외용제를 찾는 환자에게 다음 중 어떤 경우 의사 상담을 받도록 권유하십니까? (복수응답 가능)

□ 삼출성 진물이 나는 경우
□ 중등도 이상의 피부질환인 경우
□ 피부감염으로 보이는 증상이 나타나는 경우
□ 신생아 및 영아(24개월 미만) 의 경우 (기저귀발진경우 제외)
□ 체표면적의 상당 부분에 스테로이드외용제를 사용하고 있는 경우
□ 기타________________________________

14. 비처방 일반의약품 스테로이드외용제를 사용하는 환자에게 연속으로 매일 사용시 일반적으로 최대 며칠까지 사용할 수 있다고 설명하십니까?

최대 ________일

15. 스테로이드외용제 복약지도시 복약지도 장애요인이 있다고 생각하십니까?

□ 예 (15-a 문항으로 이동) □ 아니오 (16번 문항으로 이동)

15-a. 복약지도 장애요인 2가지를 선택하여 주십시오.

□ 복약지도 자료 부족
□ 복약지도를 위한 시간부족
□ 의사들의 약사 복약지도에 대한 부정적 인식
□ 환자들의 스테로이드외용제에 대한 부정적 인식
□ 환자가 스테로이드외용제에 대해 잘 알고 있을 것이라 짐작
□ 기타____

III. 환자의 스테로이드외용제 지식에 대한 약사의 생각

16. 스테로이드외용제에 관하여 환자가 어느정도 알고 있다고 생각하십니까?

		예	아니오
**16-a.**	**스테로이드외용제임**을 알고 있다		
**16-b.**	**효능** 및 **효과**에 대해 알고 있다		
**16-c.**	**세기(역가)**에 대해 알고 있다		
**16-d.**	**[비처방의약품** 사용 환자의 경우**]** **부작용**에 대해 알고 있다		
**16-e.**	**[처방의약품** 사용 환자의 경우**]** **부작용**에 대해 알고 있다		
**16-f.**	**부작용** 발생시 **대처법**에 대해 알고 있다		
**16-g.**	**사용방법** (도포량, 사용기간, 사용횟수, 제형에 따른 적용부위)을 알고 준수한다		
**16-h.**	치료 이후 **남은 스테로이드외용제**의 **저장** 및 **적용 시** 주의사항을 알고 준수한다<br>(예: 의약품을 원래 용기에서 꺼내어 다른 용기에 보관하지 않는다<br>남은 외용제를 임으로 다른 질환에 사용하지 않는다)		

17. 다음 중 스테로이드외용제에 관하여 환자의 주된 정보획득 경로라고 생각되는 3가지를 선택하여 주십시오.

□ 약사의 설명 □제품설명서 □인터넷 □ 친구(지인)
□ 의사의 설명 □광고, 신문 또는 전문잡지 □병원 또는 약국의 안내서
□ 기타___________

IV. 스테로이드외용제 부작용 실태

18. 귀하의 약국에서 스테로이드외용제 사용 후 <u>부작용 발생</u>으로 <u>약국에 호소 (방문 및 전화연락 포함) 한 환자</u>가 있습니까?

□ 예 (18-a 로 이동)

□ 아니오 (19번 문항으로 이동)

18-a. **<u>비처방</u> <u>일반</u>**의약품 스테로이드외용제 사용자 중 1개월 평균 ______%의 환자가 부작용을 호소했다.

18-b. **i. <u>처방</u>**된 **<u>일반</u>**의약품 스테로이드외용제 사용자 중 1개월 평균 ______%의 환자가 부작용을 호소했다.

ii. <u>처방</u>된 **<u>전문</u>**의약품 스테로이드외용제 사용자 중 1개월 평균 ______%의 환자가 부작용을 호소했다.

19. 스테로이드외용제로 인한 부작용이 발생한 원인으로 가능성이 높다고 생각하는 요인을 빈도순으로 기재하여 주십시오. (참고: 가장 가능성 <u>높은</u> 요인에 '1' 부여)

[ ] 약물 오용 (예: 비해당질환에 사용)
[ ] 약물 자체 요인/특성 (예: 세기/역가)
[ ] 환자 특성 (예: 노인, 만성질환, 유·소아)
[ ] 약물 남용 (예: 환자 임의 사용, 지시사항 미준수)
[ ] 기타 ________________________

20. 환자가 빈번하게 호소하는 스테로이드외용제의 부작용을 <u>3가지</u> 선택하여 주십시오.

□ 피부건조, 가려움, 자극감
□ 자반 (멍)
□ 여드름, 모낭염
□ 다모증 (털 증가)
□ 전신부작용 (안증상 포함)
□ 기타 피부부작용
□ 모세혈관확장
□ 피부위축, 살트임 (선조)
□ 피부색변화 (탈색, 색소침착)
□ 안면홍조, 주사, 입주위피부염
□ 피부감염 (세균, 진균, 바이러스)

21. 스테로이드외용제를 사용하는 환자가 부작용을 호소하는 경우 어떤 조치를 취하십니까? (복수응답 가능)

□ 투약 중단 및 병원 방문 권고
□ 지역의약품안전센터에 유해사례 보고
□ 환자가 복약지도사항을 준수하였는지 확인 후 재교육 및 재투약 권고
□ 기타__________________________

22. 아래 3가지 스테로이드외용제를 세기(역가)에 따라 순위를 부여하여 주십시오.
(참고: 세기(역가)가 높은 약물에 '1' 부여, 세기(역가)가 낮은 약물에 '3' 부여)

스테로이드외용제	세기(역가) 순위
**락티케어에취씨로션1%®** (hydrocortisone 10mg/ml, 로션제)	[ ]
**더마톱크림0.25%®** (prednicarbate 2.5㎎/g, 크림제)	[ ]
**더모베이트연고®** (clobetasol-17-propionate 0.5㎎/g, 연고제)	[ ]

설문에 끝까지 응해주셔서 감사합니다.
여러분의 의견을 소중한 정보로 사용하여 국민보건 향상에 기여할 수 있도록 하겠습니다.
