# Supplementary material for: Community pharmacists’ knowledge, perceptions, and practices about topical corticosteroid counseling: A real-world cross-sectional survey and focus group discussions in Korea
Source: PLoS One. 2020 Jul 29;15(7):e0236797. doi: 10.1371/journal.pone.0236797 (PMC7390350; doi:10.1371/journal.pone.0236797)
Supplement: Attachment — Submitted filename: Response to reviewers.docx [file pone.0236797.s007.docx]

**Response to reviewers**

Reviewer #1

- Line 35: the word “influence” has a causal meaning implying that the paper studied the causal effect of CPs’ knowledge and perceptions on the counseling practice, but the paper only examined the association under the design of cross-sectional study. Since correlation does not imply causation, the word with causal meaning like “influence”, “affect” should be replaced with “association”, “correlation”, etc., when talking about the results of statistical analysis in this study. The same issues were found in line 40, 42, 43, 45, 46, 83, 161, 206, 259, 260, 274, 294, 296.

**Thank you for pointing it out! This point is very detailed and delicate.**

**We’ve revised all of the manuscript with more proper word (associate or association) so that we can deliver the context more precisely.**

- Line 80-81: While it was true that “no research to date has investigated to what extent pharmacists contribute to patients’ safe and appropriate use of TCs”, the evidences in this study were insufficient to show how much CPs contributed to the appropriate use of TCs because reliable outcomes of TC treatment and adherence were not measured. To evaluate the effectiveness of CPs’ practices, a more analytic approach like a cohort study is needed.

**Thank you for pointing it out! We agree that revised manuscript is more precise and accurate. Actually, our study is based on the design of cross-sectional study and we can’t evaluate or measure the effectiveness of CP’s counseling practice. Therefore, we have deleted the sentence (“To our knowledge, no research to date has investigated to what extent pharmacists contribute to patients’ safe and appropriate use of TCs.”) and revised as the following.**

**“Although pharmacists are important role in patient's safe and appropriate use of TCs, research on the pharmacist's counseling behavior for TCs is very lacking.”**

- Line 105-106: Please put the figure caption below the corresponding figure at the end of paper.

**Thank you for pointing it out! We have tried to meet PLOS ONE's style requirements. Maybe, we misunderstood the PLOS ONE style templates.**

**(https://journals.plos.org/plosone/s/file?id=wjVg/PLOSOne_formatting_sample_main_body.pdf)**

- **Figure files should be saved as “Fig1.tif”, “Fig2.eps”, etc.**
- **Each figure caption should appear directly after the paragraph in which they are first cited.**

**We will check PLOS ONE guidelines for figures and revise the location of figure caption according to the reviewer comment.**

- Line 146-147: How widely was this community pharmacy dispensary software used in South Korea when this study was conducted?

**The community pharmacy dispensary software administered our survey occupied about half of all registered pharmacies at 2015. This distribution maintains similar at present. Therefore, We’ve added the following phrase (“about half of all registered pharmacies”) to clarify the meaning according to the reviewer’s comment.**

- Line 184: How many CPs were asked to do the survey? What is the response rate?

**Our survey was made to participate voluntarily through the banner when accessing the community pharmacy dispensary software. We collected the questionnaire in this way during six weeks. In fact, this program was being used by about 10,000 pharmacies, and the voluntary response could be estimated at 12.3%. We think that this is a different concept from the response rate, but it needs to be explained. Therefore, we revised the related explanation in the method and result.**

**Thank you for pointing it out! We agree that our revised manuscript is more precise by this point.**

- Line 187: Please put the figure caption below the corresponding figure at the end of paper.

**Thank you for pointing it out! We have tried to meet PLOS ONE's style requirements. Maybe, we misunderstood the PLOS ONE style templates.**

**(https://journals.plos.org/plosone/s/file?id=wjVg/PLOSOne_formatting_sample_main_body.pdf)**

- **Figure files should be saved as “Fig1.tif”, “Fig2.eps”, etc.**
- **Each figure caption should appear directly after the paragraph in which they are first cited.**

**We will check PLOS ONE guidelines for figures and revise the location of figure caption according to the reviewer comment.**

- Line 219-221: Was statistical analysis done to conclude that “Slightly more time was invested in counseling a patient on non-prescribed TCs compared with prescribed TCs”? If so, please include the test results like p-value.

**Thank you for pointing it out. We have added p-value so that we can deliver the context more precisely. We appreciate your considerate comments with details.**

- Line 254: Please put the figure caption below the corresponding figure at the end of paper.

**Thank you for pointing it out! We have tried to meet PLOS ONE's style requirements. Maybe, we misunderstood the PLOS ONE style templates.**

**(https://journals.plos.org/plosone/s/file?id=wjVg/PLOSOne_formatting_sample_main_body.pdf)**

- **Figure files should be saved as “Fig1.tif”, “Fig2.eps”, etc.**
- **Each figure caption should appear directly after the paragraph in which they are first cited.**

**We will check PLOS ONE guidelines for figures and revise the location of figure caption according to the reviewer comment.**

- Line 292: The author claimed that “this study was the first to explore CPs’ counseling practices around TC use”, but Wing Man Lau published a paper in 2017 on the CPs’ counseling practices around TC use in UK titled “Knowledge, Attitude and Advice-Giving Behavior of Community Pharmacists Regarding Topical Corticosteroids”.

**Thank you for pointing it out. We continued to review the literature while we were writing the paper, but we did not find this study. This point is very detailed and delicate. We appreciate your detailed review to catch this part.**

**We have revised the manuscript according to the reviewer’s previous comments in the part of introduction (L80-81) and added this paper in the reference (No 19). In addition, we have deleted the wrong word (the first) and added to the following sentence in the first paragraph of discussion.**

**“In the previous study conducted by Lau WM et al, there was a significant correlation between pharmacists’ attitudes toward information provision and their self-reported counselling behavior on most topics except for those related to corticosteroid safety where less advice was given.”**

- Line 375: The authors should elaborate more on the limitation of the study. The internal validity of the study heavily depended on how CPs’ practice levels were measured. Instead of objective measurement, the CPs were asked to rate their own counseling practices. As a result, the CPs’ practice quality measurement might be overestimated. In addition, the majority of CPs were surveyed through a very popular pharmacy dispensary software, which may be the source of selection bias. If possible, the authors should also discuss what had been done to reduce those biases

**Thank you for pointing it out! We agree that our revised manuscript is more precise by this point. We have tried to revise the manuscript including more detailed limitation of the study.**

**We added to following sentences in the last paragraph of discussion part.**

**“We have assessed as scored levels to minimize the measurement limits for these self-reporting practices, but this should still take into account the possibility of over-estimated quality measurements. ………..While the survey in this study has the advantage of being able to collect large numbers of samples, there is concern about the possibility of selection bias because most CPs use the same pharmacy dispensary software. To minimize the concern, we have fully recruited 1.4 times more than the initial sample, and as shown in Table 1, we have conducted the analysis after confirming that the age, gender, region, and experience of the respondents are evenly distributed. For all these efforts, this study does not directly measure the outcome for the safe use of TCs, so it should not make the error of over-interpreting the results. Finally, the interpretation and application of our findings should be limited to identifying influencing factors and exploring ways to improve the counseling practices of CPs on TCs. “**

**Once again, we truly appreciate your detailed review and comments that help improving our manuscript tremendously.**
