# Supplementary material for: Community pharmacists’ knowledge, perceptions, and practices about topical corticosteroid counseling: A real-world cross-sectional survey and focus group discussions in Korea
Source: PLoS One. 2020 Jul 29;15(7):e0236797. doi: 10.1371/journal.pone.0236797 (PMC7390350; doi:10.1371/journal.pone.0236797)
Supplement: Peer review history

# Peer review: Decision Letter 0

**DOI:** 10.1371/journal.pone.0236797.r001

**Authors:** Kevin Lu

6 Apr 2020

PONE-D-19-34348

Community pharmacists’ knowledge, perceptions, and practices about topical corticosteroid counseling: a real-world cross-sectional survey and focus group discussions in Korea

PLOS ONE

Dear Mrs. Choi,

Thank you for submitting your manuscript to PLOS ONE. After careful consideration, we feel that it has merit but does not fully meet PLOS ONE’s publication criteria as it currently stands. Therefore, we invite you to submit a revised version of the manuscript that addresses the points raised during the review process.

We would appreciate receiving your revised manuscript by May 21 2020 11:59PM. When you are ready to submit your revision, log on to https://www.editorialmanager.com/pone/ and select the 'Submissions Needing Revision' folder to locate your manuscript file.

If you would like to make changes to your financial disclosure, please include your updated statement in your cover letter.

To enhance the reproducibility of your results, we recommend that if applicable you deposit your laboratory protocols in protocols.io, where a protocol can be assigned its own identifier (DOI) such that it can be cited independently in the future. For instructions see: http://journals.plos.org/plosone/s/submission-guidelines#loc-laboratory-protocols

Please include the following items when submitting your revised manuscript:

- A rebuttal letter that responds to each point raised by the academic editor and reviewer(s). This letter should be uploaded as separate file and labeled 'Response to Reviewers'.
- A marked-up copy of your manuscript that highlights changes made to the original version. This file should be uploaded as separate file and labeled 'Revised Manuscript with Track Changes'.
- An unmarked version of your revised paper without tracked changes. This file should be uploaded as separate file and labeled 'Manuscript'.

Please note while forming your response, if your article is accepted, you may have the opportunity to make the peer review history publicly available. The record will include editor decision letters (with reviews) and your responses to reviewer comments. If eligible, we will contact you to opt in or out.

We look forward to receiving your revised manuscript.

Kind regards,

Kevin Lu, PhD

Academic Editor

Journal requirements:

When submitting your revision, we need you to address these additional requirements.

1. Please ensure that your manuscript meets PLOS ONE's style requirements, including those for file naming. The PLOS ONE style templates can be found at

https://journals.plos.org/plosone/s/file?id=wjVg/PLOSOne_formatting_sample_main_body.pdf and

https://journals.plos.org/plosone/s/file?id=ba62/PLOSOne_formatting_sample_title_authors_affiliations.pdf

2. Please include additional information regarding the survey or questionnaire used in the study and ensure that you have provided sufficient details that others could replicate the analyses. For instance, if you developed a questionnaire as part of this study and it is not under a copyright more restrictive than CC-BY, please include a copy, in both the original language and English, as Supporting Information.

3. When reporting the results of qualitative research, we suggest consulting the COREQ guidelines: http://intqhc.oxfordjournals.org/content/19/6/349. In this case, please consider including more information on the number of interviewers, their training and characteristics; how participants were selected,  and whether any inclusion and exclusion criteria were used; and how data was coded .

4. Thank you for stating the following in the Competing Interests/Financial Disclosure section:

"The authors have declared that no competing interests exist."

We note that one or more of the authors are employed by a commercial company: Wellmarker Bio Co,

1. Please provide an amended Funding Statement declaring this commercial affiliation, as well as a statement regarding the Role of Funders in your study. If the funding organization did not play a role in the study design, data collection and analysis, decision to publish, or preparation of the manuscript and only provided financial support in the form of authors' salaries and/or research materials, please review your statements relating to the author contributions, and ensure you have specifically and accurately indicated the role(s) that these authors had in your study. You can update author roles in the Author Contributions section of the online submission form.

Please also include the following statement within your amended Funding Statement.

“The funder provided support in the form of salaries for authors [insert relevant initials], but did not have any additional role in the study design, data collection and analysis, decision to publish, or preparation of the manuscript. The specific roles of these authors are articulated in the ‘author contributions’ section.”

If your commercial affiliation did play a role in your study, please state and explain this role within your updated Funding Statement.

2. Please also provide an updated Competing Interests Statement declaring this commercial affiliation along with any other relevant declarations relating to employment, consultancy, patents, products in development, or marketed products, etc.  

Within your Competing Interests Statement, please confirm that this commercial affiliation does not alter your adherence to all PLOS ONE policies on sharing data and materials by including the following statement: "This does not alter our adherence to  PLOS ONE policies on sharing data and materials.” (as detailed online in our guide for authors http://journals.plos.org/plosone/s/competing-interests) . If this adherence statement is not accurate and  there are restrictions on sharing of data and/or materials, please state these. Please note that we cannot proceed with consideration of your article until this information has been declared.

Please include both an updated Funding Statement and Competing Interests Statement in your cover letter. We will change the online submission form on your behalf.

Please know it is PLOS ONE policy for corresponding authors to declare, on behalf of all authors, all potential competing interests for the purposes of transparency. PLOS defines a competing interest as anything that interferes with, or could reasonably be perceived as interfering with, the full and objective presentation, peer review, editorial decision-making, or publication of research or non-research articles submitted to one of the journals. Competing interests can be financial or non-financial, professional, or personal. Competing interests can arise in relationship to an organization or another person. Please follow this link to our website for more details on competing interests: http://journals.plos.org/plosone/s/competing-interests

3. We note that you have indicated that data from this study are available upon request. PLOS only allows data to be available upon request if there are legal or ethical restrictions on sharing data publicly. For information on unacceptable data access restrictions, please see http://journals.plos.org/plosone/s/data-availability#loc-unacceptable-data-access-restrictions.

In your revised cover letter, please address the following prompts:

a) If there are ethical or legal restrictions on sharing a de-identified data set, please explain them in detail (e.g., data contain potentially identifying or sensitive patient information) and who has imposed them (e.g., an ethics committee). Please also provide contact information for a data access committee, ethics committee, or other institutional body to which data requests may be sent.

b) If there are no restrictions, please upload the minimal anonymized data set necessary to replicate your study findings as either Supporting Information files or to a stable, public repository and provide us with the relevant URLs, DOIs, or accession numbers. Please see http://www.bmj.com/content/340/bmj.c181.long for guidelines on how to de-identify and prepare clinical data for publication. For a list of acceptable repositories, please see http://journals.plos.org/plosone/s/data-availability#loc-recommended-repositories.

We will update your Data Availability statement on your behalf to reflect the information you provide.

Reviewers' comments:

Reviewer's Responses to Questions

**Comments to the Author**

1. Is the manuscript technically sound, and do the data support the conclusions?

The manuscript must describe a technically sound piece of scientific research with data that supports the conclusions. Experiments must have been conducted rigorously, with appropriate controls, replication, and sample sizes. The conclusions must be drawn appropriately based on the data presented. 

Reviewer #1: Yes

**********

2. Has the statistical analysis been performed appropriately and rigorously? 

Reviewer #1: Yes

**********

3. Have the authors made all data underlying the findings in their manuscript fully available?

The PLOS Data policy requires authors to make all data underlying the findings described in their manuscript fully available without restriction, with rare exception (please refer to the Data Availability Statement in the manuscript PDF file). The data should be provided as part of the manuscript or its supporting information, or deposited to a public repository. For example, in addition to summary statistics, the data points behind means, medians and variance measures should be available. If there are restrictions on publicly sharing data—e.g. participant privacy or use of data from a third party—those must be specified.

Reviewer #1: No

**********

4. Is the manuscript presented in an intelligible fashion and written in standard English?

PLOS ONE does not copyedit accepted manuscripts, so the language in submitted articles must be clear, correct, and unambiguous. Any typographical or grammatical errors should be corrected at revision, so please note any specific errors here.

Reviewer #1: Yes

**********

5. Review Comments to the Author

Please use the space provided to explain your answers to the questions above. You may also include additional comments for the author, including concerns about dual publication, research ethics, or publication ethics. (Please upload your review as an attachment if it exceeds 20,000 characters)

Reviewer #1: Thank you for the opportunity to review this paper. This paper discusses an important topic and has investigated the relationship between community pharmacist (CP) counseling behavior and factors related to CPs’ knowledge, perception, and other characteristics, and provided suggestions on how CP can promote safe use of topical corticosteroids (TCs). The paper is well written, and the research topic is important as CPS are well placed to educate patients who use TCs to improve medication adherence. Below are a few comments for the authors to consider.

**********

6. PLOS authors have the option to publish the peer review history of their article (what does this mean?). If published, this will include your full peer review and any attached files.

If you choose “no”, your identity will remain anonymous but your review may still be made public.

**Do you want your identity to be public for this peer review?** For information about this choice, including consent withdrawal, please see our Privacy Policy.

Reviewer #1: No

[NOTE: If reviewer comments were submitted as an attachment file, they will be attached to this email and accessible via the submission site. Please log into your account, locate the manuscript record, and check for the action link "View Attachments". If this link does not appear, there are no attachment files to be viewed.]

While revising your submission, please upload your figure files to the Preflight Analysis and Conversion Engine (PACE) digital diagnostic tool, https://pacev2.apexcovantage.com/. PACE helps ensure that figures meet PLOS requirements. To use PACE, you must first register as a user. Registration is free. Then, login and navigate to the UPLOAD tab, where you will find detailed instructions on how to use the tool. If you encounter any issues or have any questions when using PACE, please email us at figures@plos.org. Please note that Supporting Information files do not need this step.

# Author comment: Author response to Decision Letter 0

**DOI:** 10.1371/journal.pone.0236797.r002

26 Jun 2020

Reviewer #1

- Line 35: the word “influence” has a causal meaning implying that the paper studied the causal effect of CPs’ knowledge and perceptions on the counseling practice, but the paper only examined the association under the design of cross-sectional study. Since correlation does not imply causation, the word with causal meaning like “influence”, “affect” should be replaced with “association”, “correlation”, etc., when talking about the results of statistical analysis in this study. The same issues were found in line 40, 42, 43, 45, 46, 83, 161, 206, 259, 260, 274, 294, 296.

Thank you for pointing it out! This point is very detailed and delicate.

We’ve revised all of the manuscript with more proper word (associate or association) so that we can deliver the context more precisely. 

- Line 80-81: While it was true that “no research to date has investigated to what extent pharmacists contribute to patients’ safe and appropriate use of TCs”, the evidences in this study were insufficient to show how much CPs contributed to the appropriate use of TCs because reliable outcomes of TC treatment and adherence were not measured. To evaluate the effectiveness of CPs’ practices, a more analytic approach like a cohort study is needed.

Thank you for pointing it out! We agree that revised manuscript is more precise and accurate. Actually, our study is based on the design of cross-sectional study and we can’t evaluate or measure the effectiveness of CP’s counseling practice. Therefore, we have deleted the sentence (“To our knowledge, no research to date has investigated to what extent pharmacists contribute to patients’ safe and appropriate use of TCs.”) and revised as the following.

“Although pharmacists are important role in patient's safe and appropriate use of TCs, research on the pharmacist's counseling behavior for TCs is very lacking.”

- Line 105-106: Please put the figure caption below the corresponding figure at the end of paper.

Thank you for pointing it out! We have tried to meet PLOS ONE's style requirements. Maybe, we misunderstood the PLOS ONE style templates. 

(https://journals.plos.org/plosone/s/file?id=wjVg/PLOSOne_formatting_sample_main_body.pdf)

• Figure files should be saved as “Fig1.tif”, “Fig2.eps”, etc.

• Each figure caption should appear directly after the paragraph in which they are first cited.

We will check PLOS ONE guidelines for figures and revise the location of figure caption according to the reviewer comment.

- Line 146-147: How widely was this community pharmacy dispensary software used in South Korea when this study was conducted?

The community pharmacy dispensary software administered our survey occupied about half of all registered pharmacies at 2015. This distribution maintains similar at present. Therefore, We’ve added the following phrase (“about half of all registered pharmacies”) to clarify the meaning according to the reviewer’s comment.

- Line 184: How many CPs were asked to do the survey? What is the response rate?

Our survey was made to participate voluntarily through the banner when accessing the community pharmacy dispensary software. We collected the questionnaire in this way during six weeks. In fact, this program was being used by about 10,000 pharmacies, and the voluntary response could be estimated at 12.3%. We think that this is a different concept from the response rate, but it needs to be explained. Therefore, we revised the related explanation in the method and result.

Thank you for pointing it out! We agree that our revised manuscript is more precise by this point. 

- Line 187: Please put the figure caption below the corresponding figure at the end of paper.

Thank you for pointing it out! We have tried to meet PLOS ONE's style requirements. Maybe, we misunderstood the PLOS ONE style templates. 

(https://journals.plos.org/plosone/s/file?id=wjVg/PLOSOne_formatting_sample_main_body.pdf)

• Figure files should be saved as “Fig1.tif”, “Fig2.eps”, etc.

• Each figure caption should appear directly after the paragraph in which they are first cited.

We will check PLOS ONE guidelines for figures and revise the location of figure caption according to the reviewer comment.

- Line 219-221: Was statistical analysis done to conclude that “Slightly more time was invested in counseling a patient on non-prescribed TCs compared with prescribed TCs”? If so, please include the test results like p-value.

Thank you for pointing it out. We have added p-value so that we can deliver the context more precisely. We appreciate your considerate comments with details. 

- Line 254: Please put the figure caption below the corresponding figure at the end of paper.

Thank you for pointing it out! We have tried to meet PLOS ONE's style requirements. Maybe, we misunderstood the PLOS ONE style templates. 

(https://journals.plos.org/plosone/s/file?id=wjVg/PLOSOne_formatting_sample_main_body.pdf)

• Figure files should be saved as “Fig1.tif”, “Fig2.eps”, etc.

• Each figure caption should appear directly after the paragraph in which they are first cited.

We will check PLOS ONE guidelines for figures and revise the location of figure caption according to the reviewer comment.

- Line 292: The author claimed that “this study was the first to explore CPs’ counseling practices around TC use”, but Wing Man Lau published a paper in 2017 on the CPs’ counseling practices around TC use in UK titled “Knowledge, Attitude and Advice-Giving Behavior of Community Pharmacists Regarding Topical Corticosteroids”.

Thank you for pointing it out. We continued to review the literature while we were writing the paper, but we did not find this study. This point is very detailed and delicate. We appreciate your detailed review to catch this part.

We have revised the manuscript according to the reviewer’s previous comments in the part of introduction (L80-81) and added this paper in the reference (No 19). In addition, we have deleted the wrong word (the first) and added to the following sentence in the first paragraph of discussion.

“In the previous study conducted by Lau WM et al, there was a significant correlation between pharmacists’ attitudes toward information provision and their self-reported counselling behavior on most topics except for those related to corticosteroid safety where less advice was given.” 

- Line 375: The authors should elaborate more on the limitation of the study. The internal validity of the study heavily depended on how CPs’ practice levels were measured. Instead of objective measurement, the CPs were asked to rate their own counseling practices. As a result, the CPs’ practice quality measurement might be overestimated. In addition, the majority of CPs were surveyed through a very popular pharmacy dispensary software, which may be the source of selection bias. If possible, the authors should also discuss what had been done to reduce those biases

Thank you for pointing it out! We agree that our revised manuscript is more precise by this point. We have tried to revise the manuscript including more detailed limitation of the study.

We added to following sentences in the last paragraph of discussion part.

“We have assessed as scored levels to minimize the measurement limits for these self-reporting practices, but this should still take into account the possibility of over-estimated quality measurements. ………..While the survey in this study has the advantage of being able to collect large numbers of samples, there is concern about the possibility of selection bias because most CPs use the same pharmacy dispensary software. To minimize the concern, we have fully recruited 1.4 times more than the initial sample, and as shown in Table 1, we have conducted the analysis after confirming that the age, gender, region, and experience of the respondents are evenly distributed. For all these efforts, this study does not directly measure the outcome for the safe use of TCs, so it should not make the error of over-interpreting the results. Finally, the interpretation and application of our findings should be limited to identifying influencing factors and exploring ways to improve the counseling practices of CPs on TCs. “

Once again, we truly appreciate your detailed review and comments that help improving our manuscript tremendously

Attachment Submitted filename: Response to reviewers.docx Click here for additional data file.

# Editor report: Decision Letter 1

**DOI:** 10.1371/journal.pone.0236797.r003

**Authors:** Kevin Lu

15 Jul 2020

Community pharmacists’ knowledge, perceptions, and practices about topical corticosteroid counseling: a real-world cross-sectional survey and focus group discussions in Korea

PONE-D-19-34348R1

Dear Dr. Choi,

We’re pleased to inform you that your manuscript has been judged scientifically suitable for publication and will be formally accepted for publication once it meets all outstanding technical requirements.

Within one week, you’ll receive an e-mail detailing the required amendments. When these have been addressed, you’ll receive a formal acceptance letter and your manuscript will be scheduled for publication.

An invoice for payment will follow shortly after the formal acceptance. To ensure an efficient process, please log into Editorial Manager at http://www.editorialmanager.com/pone/, click the 'Update My Information' link at the top of the page, and double check that your user information is up-to-date. If you have any billing related questions, please contact our Author Billing department directly at authorbilling@plos.org.

If your institution or institutions have a press office, please notify them about your upcoming paper to help maximize its impact. If they’ll be preparing press materials, please inform our press team as soon as possible -- no later than 48 hours after receiving the formal acceptance. Your manuscript will remain under strict press embargo until 2 pm Eastern Time on the date of publication. For more information, please contact onepress@plos.org.

Kind regards,

Kevin Lu, PhD

Academic Editor

PLOS ONE

# Editor report: Acceptance letter

**DOI:** 10.1371/journal.pone.0236797.r004

**Authors:** Kevin Lu

17 Jul 2020

PONE-D-19-34348R1 

Community pharmacists’ knowledge, perceptions, and practices about topical corticosteroid counseling: a real-world cross-sectional survey and focus group discussions in Korea 

Dear Dr. Choi:

I'm pleased to inform you that your manuscript has been deemed suitable for publication in PLOS ONE. Congratulations! Your manuscript is now with our production department. 

If your institution or institutions have a press office, please let them know about your upcoming paper now to help maximize its impact. If they'll be preparing press materials, please inform our press team within the next 48 hours. Your manuscript will remain under strict press embargo until 2 pm Eastern Time on the date of publication. For more information please contact onepress@plos.org.

If we can help with anything else, please email us at plosone@plos.org. 

Thank you for submitting your work to PLOS ONE and supporting open access. 

Kind regards, 

PLOS ONE Editorial Office Staff

on behalf of

Professor Kevin Lu 

Academic Editor

PLOS ONE
